# Costs of obesity attributable to the consumption of sugar-sweetened beverages in Brazil

**DOI:** 10.1038/s41598-024-59991-8

**Published:** 2024-06-17

**Authors:** Bruna Farias da Silva, Adélia da Costa Pereira de Arruda Neta, Rômulo Eufrosino de Alencar Rodrigues, Jevuks Matheus de Araújo, Patrícia Vasconcelos Leitão Moreira, Flávia Emília Leite Lima Ferreira, Rodrigo Pinheiro de Toledo Vianna, José Moreira da Silva Neto, Eduardo de Carli, Rafaela Lira Formiga Cavalcanti de Lima

**Affiliations:** 1 Postgraduate Program in Nutrition Science, Federal University of Paraíba, João Pessoa, Paraíba Brazil; 2 Núcleo de Pesquisas em Alimentação-NEPS, Universidade Estadual de Campinas, São Paulo, Brazil; 3 Department of Economics, Federal University of Paraíba, João Pessoa, Paraíba Brazil; 4 Department of Nutrition, Federal University of Paraíba, João Pessoa, Paraíba Brazil; 5 Technical Health School, Federal University of Paraíba, João Pessoa, Paraíba Brazil; 6 Department of Nutrition, School of Public Health. University of São Paulo, São Paulo, Brazil

**Keywords:** Diseases, Risk factors

## Abstract

Excess sugar is considered one of the primary factors contributing to overweight status. In Brazil, sugar-sweetened beverages (SSBs) contain a significant amount of this nutrient and are consumed excessively. These beverages are associated with adverse health outcomes and impose costs on the healthcare system. The literature currently lacks studies that aim to attribute specific nutrients or foods as causes of diseases and also evaluate their economic impact, especially in middle- and low-income countries. This study aims to estimate the direct and indirect costs of obesity, stratified by sex and age group, resulting from the excessive consumption of sugar-sweetened beverages in Brazil from 2008 to 2020, and to project these costs for the year 2036. The estimation of obesity costs attributable to excessive consumption of SSBs was based on relative risks and the population prevalence of obesity, considering expenditures on hospitalizations and outpatient procedures in the Unified Health System (SUS). Cost information was obtained from the health information systems available at SUS. The highest burden attributable to the consumption of SSBs was observed among younger individuals and progressively decreased with advancing age. The total direct costs in the period between 2008 and 2020 amounted to approximately US$ 6.33 million, 87% of which was related to expenses for females. Additionally, deaths resulting from the consumption of SSBs cost the economy US$ 40 million due to the premature loss of productivity. The total costs of obesity attributable to the consumption of SSBs are substantial, impacting public spending and generating social and productivity losses that burden the economy. It is crucial to develop and implement cost-effective fiscal and regulatory policies aimed at preventing and combating obesity.

## Introduction

Obesity is recognized as a significant public health issue due to its immediate impact on health, such as the development of non-communicable chronic diseases (NCDs), and long-term consequences, including mortality, resulting in millions of years of life lost worldwide^1–3^. The prevalence of obesity is on the rise globally, and Brazil is no exception, affecting 25.9% of its population in 2019^4^.

It is projected that by 2030, 1 in 5 women and 1 in 7 men will be living with obesity, equating to more than 1 billion people globally. The global prevalence of obesity is higher in women than in men, and the majority of individuals with obesity now reside in low- and middle-income countries^3^. These countries face the dual burden of malnutrition and obesity, where health systems are unprepared and ill-equipped to address obesity and its consequences^3^.

Obesity significantly affects an individual's life, encompassing psychological distress due to stigma and discrimination, as well as the development of metabolic complications that elevate the risk of cardiovascular diseases, type 2 diabetes mellitus, and certain types of cancer^5^.

Obesity, defined as a multifactorial chronic disease, results from various driving and determinative factors, including genetics, biology, access to healthcare, mental health, sociocultural influences, economic conditions, consumption of ultra-processed foods, and environmental determinants (e.g., food advertisingg). These factors interact with one another, contributing to the disease's development^3^. Epidemiological studies have underscored the significance of diet in the etiology of obesity and its comorbid conditions^6–8^.

Currently, dietary analysis often focuses on the level of food processing, utilizing the widely recognized NOVA classification that categorizes foods into four groups: (1) unprocessed or minimally processed foods (obtained in their same or nearly natural state, such as fresh fruits or packaged rice); (2) culinary ingredients (derived from unprocessed foods and used in cooking, such as oils, salt, and sugar); (3) processed foods (combinations of the first two groups, like strawberry jam made from fruit with added sugar); and (4) ultra-processed foods, which are beverages or products not traditionally recognized as foods but rather formulations of substances derived from the fractionation of foods from the first category^9^. Among these categories, ultra-processed foods have been identified as having a more detrimental impact on health, being associated with increased risks of obesity, diabetes, and other non-communicable chronic diseases, as well as depression and dementia^9–16^.

Sweetened beverages (soft drinks, artificial juices, teas, among others) are among the most consumed food groups in Brazil, with an average consumption of 65 L/year per individual^17,18^. These products contain excessive amounts of sugar, are high in calories, have low nutritional value, and contribute little to satiety^19,20^. It is worth noting that excess sugar is considered one of the main causes of excess weight and, consequently, its associated diseases (type 2 diabetes, hypertension)^20,21^. Therefore, the consumption of sugar-sweetened beverages is associated with an increased risk of developing obesity^20^.

There remains a scarcity of literature that connects the consumption of a specific nutrient or food to the causation of disease while also assessing its economic impact, particularly in low- and middle-income countries^22–24^. Although the etiology of obesity is multifaceted, excessive sugar intake is deemed a significant contributor to overweight and related diseases^21^. Health systems are economically strained due to excessive expenditures; for example, the amount allocated by the Unified Health System (SUS) for the care of overweight and obesity in 2019 was R$1.5 billion, accounting for 22% of the annual direct expenditures on non-communicable diseases (NCDs) in Brazil^25^. Consequently, it is crucial to conduct studies to ascertain the cost of illnesses, which can shed light on the burden on the health system and the economy. Such studies can also provide a theoretical basis for the enhancement and formulation of effective regulatory public policies aimed at reducing sugar consumption, preventing diet-related health issues, and significantly impacting the socio-economic capacity of individuals to manage obesity^24^.

This study gains additional relevance in light of the current Brazilian political scenario, which seeks to debate and pass a new tax reform aimed at correcting tax distortions and inequalities. This includes the introduction of a selective tax on products and services harmful to health, to promote the production and consumption of healthy foods.

Therefore, this study aims to estimate the direct and indirect costs of obesity, stratified by sex and age group, resulting from the excessive consumption of sugar-sweetened beverages in Brazil from 2008 to 2020, and to project these costs up to 2036. We hypothesize that the consumption of sugar-sweetened beverages has an impact on obesity and, consequently, on the direct and indirect costs associated with this chronic non-communicable disease.

## Methods

### Data

Information was collected from national databases to calculate the direct and indirect costs of obesity in the period between 2008 and 2020 in Brazil. To estimate the population attributable fraction (PAF) related to the consumption of sugar-sweetened beverages, data were obtained from the Family Budget Survey (*Pesquisa de Orçamento Familiar*, POF) for the years 2008/2009 and 2017/2018, conducted by the Brazilian Institute of Geography and Statistics (IBGE). This survey provides information related to family food consumption^17,26^.

The direct cost data used in this study focused on the hospital admission and outpatient care spending of the Brazilian public health system (SUS) associated with obesity. These data were extracted from the Hospital Information System (SIH/SUS) and the Outpatient Information System (SIA/SUS), encompassing treatment costs such as medical consultations, procedures, and medications for the entire study period. The data were collected from the DATASUS database of the Brazilian Ministry of Health. DATASUS is considered a secondary, publicly accessible database. It comprises various systems and subsystems that provide information related to the Brazilian population, including mortality, hospital and outpatient care, primary care, healthcare, live births, the physical infrastructure of hospitals and outpatient clinics, healthcare professionals within the SUS, notifications of epidemiological disease outbreaks, among other data^27^.

Three databases were used for the calculation of indirect costs: the observational characteristics of deceased people between 2008 and 2020 were collected from the Mortality Information System (SIM); the income characteristics of the living for the years 2008 to 2015 were obtained from IBGE's National Household Sample Survey (PNAD); and finally, the mortality probabilities of the population by age and sex were extracted from the complete mortality tables of the IBGE for the years 2008 to 2020^28^.

In addition, to filter the data regarding obesity that resulted in hospitalizations, outpatient visits and mortality, this disease was classified according to the 10th revision of the International Classification of diseases—ICD 10, with the code E66 for obesity^29^. Furthermore, the age groups were stratified according to the Pan American Health Organization standard for both sexes: 25–34 years, 35–44 years, 55–64 years, 65–74 years and 75–84 years (it is worth noting that for indirect costs, the last age evaluated was 65 years)^30^.

All monetary values were corrected by the Brazilian Consumer Price Index (IPCA/IBGE) in Brazilian currency (R$—reais) for 2020 and subsequently converted to US dollars ($) at the average exchange rate for 2020, corresponding to 4,604 R$/U$S (021720 U$S/R$), made available by the Institute for Applied Economic Research (IPEA).

The research protocol received approval from the Ethics Committee of the Federal University of Paraíba, under consent number 3,843,739. Given the secondary nature of the data available on public domain sites through the Brazilian public health system, the Ethics Committee waived the requirement for informed consent. It is important to emphasize that the primary studies concerning the POF data collection were conducted in accordance with all relevant regulations, following the methodology described in its documentation^17,26^.

### Calculation of the population attributable fraction (PAF)

The proportion of the risk of developing obesity that could be eliminated in each period if the consumption of sugar-sweetened beverages were reduced to its theoretical minimum risk exposure level (TMREL) is known as the population attributable fraction (PAF). It is defined as^31^:

1 $$PAF=\frac{{\int }_{0}^{l}RR(x)P\left(x\right)d\left(x\right)-RR\left(x\right)TMREL}{{\int }_{0}^{l}RR(x)P\left(x\right)d\left(x\right)}$$

where P(x) represents the current distribution of food consumption, RR (x) is the relative risk of developing obesity at exposure (x), and (l) signifies the maximum level of exposure, i.e., consumption of the nutrient. TMREL stands for the theoretical minimum risk exposure level; that is, the level of consumption considered optimal for minimizing the risk at the population level. In this study, the adopted TMREL for sugar-sweetened beverage is set tat 50 kcal per serving of 226.8 g of sweetened beverages^32^.

The relative risk, RR(x), is defined as follows^33^:

2 $$\begin{gathered} {\text{exp}}\left( {{\upbeta }\left( {{\text{x }} - {\text{ y}}\left( {\text{x}} \right)} \right)} \right){\text{if x }} - {\text{ y}}\left( {\text{x}} \right){ } \ge { }0 \hfill \\ {\text{or}} \hfill \\ 1\,\, if x - y\left( x \right) < 0 \hfill \\ \end{gathered}$$

where β is the change in the log of the relative risk per unit of exposure, x is the current exposure level, and y(x) is the TMREL.

Food consumption was assessed through food diaries completed on non-consecutive days, during which participants were instructed to meticulously record the names of the foods consumed, the preparation methods, the measures used, the quantities ingested, the times of consumption, and whether the food was consumed at home or outside. Portions were converted from standard units or household measures to grams using a common reference table for each household participating in the POF survey^17^. A second assessment of food records was conducted in a sub-sample of the POF^17^. The estimation of the habitual nutrient intake was performed using the *Multiple Source Method* (MSM). This method is suitable for estimating the habitual individual intake for repeated measurements over a specified period.

To determine the distribution that best fit the sample data, a goodness-of-fit test was conducted. This test confirmed that the data on sugar-sweetened beverage consumption followed a Gamma distribution, which was subsequently used in the calculation of the PAF.

### Direct costs

The method employed to estimate the direct costs of obesity utilized the top-down approach, which assesses costs in aggregate by disease. The costs were derived from the total expenses associated with hospitalizations and outpatient care within the SUS. Public expenditures on obesity were estimated through data from both the Hospital Information System (SIH/SUS) and the Outpatient Information System (*Sistema de Informação Ambulatorial*, SIA/SUS)^27^.

The base equation is outlined as follows:

3 $${\text{TDCt}},{\text{a}},\mathrm{ b}= \sum_{{\text{n}}=1}^{{\text{n}}}\mathrm{SIH ij},{\text{t}},{\text{a}}+ \sum_{{\text{k}}=1}^{{\text{k}}}\mathrm{SIA aj},{\text{t}},{\text{a}}$$

where TDCt,a,b represents the total direct cost of all hospital admissions (SIHij,t,a) plus k outpatient care instances (SIAaj,t,a), for individuals (ij) and (aj), at time (t), in age group (a) and sex (b) for obesity. To isolate the effect attributable to the excessive intake of sugar-sweetened beverages, we weighted Eq. 3 by the PAF:

4 $${\text{TCDba}},{\text{t}},\mathrm{ a},\mathrm{ b}=(\sum_{{\text{n}}=1}^{{\text{n}}}{\text{SIHij}},{\text{t}},{\text{a}},{\text{b}}+\sum_{{\text{k}}=1}^{{\text{k}}}{\text{SIAaj}},{\text{t}},{\text{a}},{\text{b}})*{\text{PAFba}}$$

Therefore, PAFba, a, b represents the population attributable fraction by age group and sex with regard to sugar-sweetened beverages; that is, the portion of costs attributable to the excessive consumption of these beverages.

### Indirect costs

Two methods can be used to estimate the indirect costs related to the disease: *the human capital approach*, which is associated with productivity losses, and the *friction costs method*, which calculates the cost associated with replacing a sick worker; that is, it estimates the costs of worker replacement. In this study, the human capital approach was adopted, and productivity losses were estimated based on wages over the working life of an individual. Observational variables characterizing the lifetime income of living individuals were correlated with the observational characteristics of individuals who died due to obesity before the age of 65 years. This method, therefore, estimates the income of the deceased individual given the probability of their being alive.

The methodology employed in Ywata et al.^34^ was partially adopted for this study. The authors assessed the loss of human capital due to premature external deaths, such as those resulting from homicides and traffic accidents. By utilizing income data of individuals and cross-referencing this information with age, sex, education, and geographic location of residence, it is possible to derive an estimate of the average income for subgroups of the population (IBGE). Accordingly, the estimated income over time was projected to calculate the income flow for these population subgroups. To distinguish between causes, the income flow for each population subgroup was estimated based on the PAF.

The irreversible productivity losses due to the premature deaths of individuals aged 25–65 years attributable to obesity were considered in this study. To facilitate this analysis, six explanatory variables for income, as provided by the SUS and the SIM databases, were utilized: the state of the country in which the individual resides (residence), age, sex, education, color/race, and marital status.

As accurately predicting the future income of deceased persons is impossible, the data of living individuals from the PNAD database were used to match the deceased individuals with the observational variables of both databases. Thus, the income information of individuals recorded in the PNAD was applied to the deceased in the SIM database, aiming to align the six selected variables, given that income data is exclusively found in the PNAD database. The match between the databases was perfect for 83.8% of the observations. For the combinations that did not match perfectly, variables were removed according to their significance in explaining income, first removing marital status and then color/race. Given the impossibility of predicting changes in the level of education, residence situation, and marital status of deceased persons over time, only the variable of age was adjusted. For example, an individual who died at the age of 25 in 2019 had their age incremented in the following years to 26, 27, and so on, until reaching the maximum productivity age of 65 in 2049, considering the probability of survival. Hence, all other variable values remain constant over time. To discount the flow of future income to its present value, the net present value (NPV) formula was applied, which measures the value of money over time:

5 $${\text{TICt}},{\text{i}}=\sum_{{\text{X}}={\text{Di}}}^{{\text{T}}}\frac{1}{{(1+{\text{d}})}^{({\text{x}}-{\text{Di}})}}*{\text{Pr}}\left({\text{Fi}}>{\text{x}}|{\text{Fi}}\ge {\text{Di}}\right)*{\text{Wi}}$$

where (d) is the annual discount rate of 3%, (Wi) is the expected annual discount rate of income of individual (i) in the SIM database, and 'T' is the maximum productive age, in this case 65 years. The probability Pr (Fi > x|Fi Di) is the probability that the individual is alive at age (x), since he did not die at the age of (Di); that is, the age recorded in the SIM. The age of death is indicated by (Fi), given that individual (i) has already passed away. Finally, TICt, i is the total indirect cost for individual (i) at time (t). Similarly, to isolate the effect of sugar-sweetened beverages on direct costs, we get:

6 $${\text{TICba}},{\text{t}},{\text{i}}=\sum_{{\text{X}}={\text{Di}}}^{{\text{T}}}\frac{1}{{(1+{\text{d}})}^{({\text{x}}-{\text{Di}})}}*{\text{Pr}}\left({\text{Fi}}>{\text{x}}|{\text{Fi}}\ge {\text{Di}}\right)*{\text{Wi}}*{\text{PAFba}}$$

Therefore, to obtain the overall total indirect cost and the overall total attributable indirect cost, we added up the individual costs and grouped them by age group. In addition, if the individual had not died, the expected years of productive life lost to premature deaths were calculated as follows:

7 $${\text{YLLt}},{\text{i}},{\text{ba}}= \sum_{{\text{x}}={\text{Di}}}^{{\text{T}}}{\text{x}}*{\text{Pr}}({\text{Fi}}>{\text{x}}\left|{\text{Fi}}\ge {\text{Di}}\right)*{\text{PAFba}}$$

where YLLt, i, bbs are the Years of Life Lost (YLL) by the individual due to premature death from obesity attributable to excessive use of sugar-sweetened beverages. This methodological approach to estimate indirect costs was considered due to the costs associated with the permanent loss of productivity due to premature death due to obesity, caused by excessive consumption of these drinks, hypothetically being the highest within this cost modality^34^. In addition, the estimates are more realistic because real observational variables were used as determinants of the income of deceased people, reducing possible biases in the results^34^.

### Projecting direct and indirect costs

To project the trends in direct and indirect costs attributable to the consumption of sugar-sweetened beverages up to the year 2036, a time-series linear regression model was utilized. This model accounts for the non-seasonality and absence of cyclical patterns in the costs, while controlling for age groups and sex of the individuals, as illustrated below:

8 $$\begin{gathered} Y_{t} = \alpha + Trend_{t} + \beta X_{t}{\prime} + \mu_{t} \hfill \\ Y_{t + h} = \alpha + Trend_{t + h} + \beta X_{t}{\prime} + \mu_{t + h} \hfill \\ \end{gathered}$$

where Yt denotes the direct costs in year t (2008, 2009…2020) for direct costs and t (2008, 2009…2019) for indirect costs, incorporating the time trend given the vector of characteristic regressors, Xt′. Subsequently, future values of the attributable costs, Yt + h, are estimated for h = 2021, 2022…2036 for direct costs and h = 2020, 2021…2036 for indirect costs. A residual, ut + h, is also generated for each prediction. The estimates are provided with a 95% confidence interval.

## Results

Table 1 illustrates the estimated impacts of excessive consumption of sugar-sweetened beverages by age group and sex. For both genders, the highest percentages were noted in the age group of 25–34 years, with 4.1% for women and 3.7% for men during the 2017–2018 period. When comparing between sexes, the closest similarities in PAF were found in the age group from 25–34 to 65–74 years for both the 2008–2009 and 2017–2018 periods. However, an inverse correlation between PAF and age was observed in the periods analyzed. Overall, PAFs increased across all age groups and for both sexes between the years 2008–2009 and 2017–2018. Notably, in the age group of 75–84 years, the difference in men was nine-fold greater.

**Table 1:** Population attributable fraction for the consumption of sugar-sweetened beverages by sex and age, Brazil, 2023.

Age range	2008–2009		2017–2018	
	Men PAFba	Women PAFba	Men PAFba	Women PAFba
25–34	0.031	0.035	0.037	0.041
35–44	0.029	0.032	0.034	0.039
45–54	0.026	0.031	0.032	0.037
55–64	0.010	0.025	0.030	0.034
65–74	0.013	0.016	0.028	0.032
75–84	0.002	0.008	0.019	0.008
Mean	0.019	0.024	0.030	0.032

Table 2 indicates that, overall, the prevalence of obesity is higher in men and in the age groups of 35–44 years and 45–54 years in all years studied. Women have prevalence values similar to those of men, but in the age groups of 65–74 years and > 75 years the prevalence of obesity is higher when compared to men. Additionally, there is an observable upward trend in the average prevalence of obesity among both sexes over the years (2008, 2020, and 2036), with projections showing an increase from approximately 15% in 2008 to 30% in 2036.

**Table 2:** Projection of obesity prevalence for 2036. Brazil, 2023.

Age range	Men (%)			Women (%)		
	2008	2020	2036*	2008	2020	2036*
25–34	12.5	19.3	28.4	7.6	16.4	22.9
35–44	16.9	25.2	33.6	11.0	20.6	27.2
45–54	18.1	24.6	33.6	15.9	20.4	30.4
55–64	17.4	23.5	32.7	18.3	20.6	32.6
65–74	13.2	18.9	28.8	15.8	19.8	32.1
> 75	13.4	22.7	24.2	22.2	27.6	30.3
Mean	15.25	22.36	30.22	15.13	20.90	29.25

Regarding direct costs, approximately 65 thousand hospitalizations and 357 thousand outpatient visits were recorded due to obesity between 2008 and 2020. There was a predominance of women in the age group of 35–44 years. In this period, US$ 6.33 million in public resources were spent with hospitalizations and outpatient care resulting from the excessive consumption of sugar-sweetened beverages, corresponding to 87% of spending for women. In total, a decrease in direct cost between 2008 and 2020 in the order of 6% could be observed in men and an increase of 3% in women (Table 3).

**Table 3:** Direct costs with obesity attributed to the consumption of sugar-sweetened beverages by sex and age group in US$ per thousand. Brazil, 2023.

Population	Year	Age range						
		25–34	35–44	45–54	55–64	65–74	> 75	Total
Men	2008	15.066	11.041	7.257	1.116	0.221	0.014	34.717
	2020	8.973	11.986	8.368	3.164	0.265	0.059	32.817
	2036*	42.317	58.225	35.897	15.970	2.291	0.090	154.793
Women	2008	70.925	72.147	49.013	13.506	0.676	0.143	206.412
	2020	62.511	77.860	49.860	21.544	2.066	0.024	213.868
	2036*	279.098	369.059	223.468	98.210	12.356	0.013	982.192
Men + women	2008	85.991	88.901	56.270	14.622	0.897	0.157	241.130
	2020	71.484	89.847	58.228	24.708	2.332	0.084	246.686
	2036*	321.416	427.285	259.366	114.181	14.648	0.103	1137.001
Total	2008–2036	6388.366	7730.930	4749.010	1896.639	212.349	2.229	20,979.530

*Projection of direct costs for 2036.

**Figure 1:**
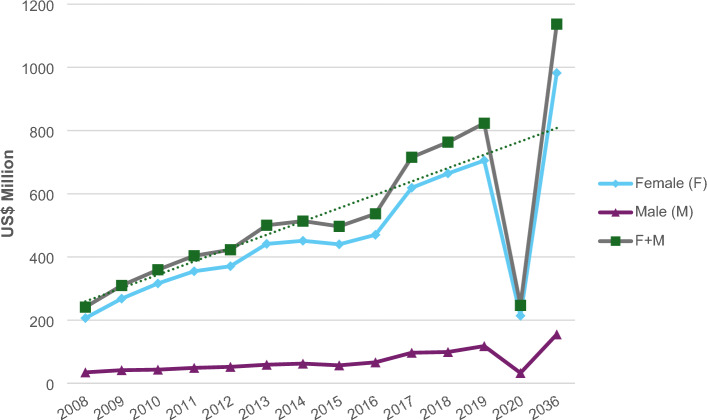
Trend for the direct costs with obesity attributed to the consumption of sugar-sweetened beverages according to the period from 2008 to 2020 and projection for 2036 in Brazil. Brazil, 2023.

In general, total direct costs grew over the years with a more significant increase after 2020 (Fig. 1). According to the projections for 2036, the trend indicates an even more pronounced growth in costs associated with hospital admissions and outpatient care.

Table 4 shows the indirect costs of premature deaths due to obesity associated with the excessive consumption of sugar-sweetened beverages. In the period from 2008 to 2020, a total of 19.1932 deaths due to obesity were recorded, of which 58% were men and aged 55–65 years. The economic cost attributed to deaths resulting from the consumption of sugar-sweetened beverages amounted to US$ 40 million, due to the premature loss of productivity, with males contributing approximately 51% to this total. For both sexes, the proportion of these costs increased between 2008 and 2020, with the highest indirect costs observed in individuals within the 35–44 year age group.

**Table 4:** Indirect costs with obesity attributed to the consumption of sugar-sweetened beverages by sex and age group in US$ per thousand. Brazil, 2023.

Population	Year	Age range				
		25–34	35–44	45–54	55–65	Total
Men	2008	248.068	381.993	310.471	65.732	1006.267
	2020	438.225	802.742	695.088	283.719	2219.776
	2036*	655.040	1456.272	1172.171	647.541	3931.026
Women	2008	210.403	326.939	396.748	164.436	1098.528
	2020	348.577	618.020	597.440	294.068	1858.106
	2036*	457.387	1008.743	917.410	466.260	2849.802
Men + women	2008	458.472	708.932	707.220	230.169	2104.795
	2020	786.803	1420.762	1292.528	577.787	4077.882
	2036*	1112.428	2465.016	2089.582	1113.801	6780.827
Total	2008–2036	23,997.680	44,987.540	40,372.640	18,698.900	128,056.760

*Projection of indirect costs for 2036.

**Figure 2:**
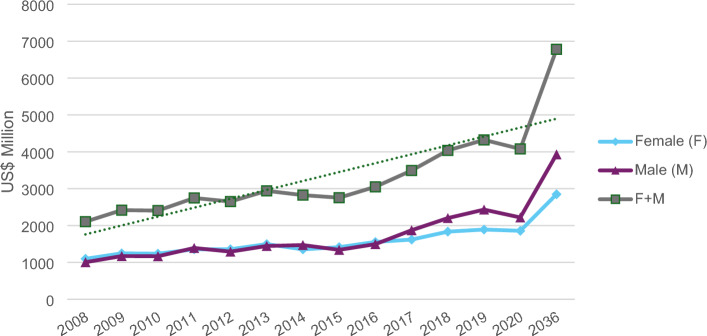
Trend for the indirect costs with obesity attributed to the consumption of sugar-sweetened beverages according to the period from 2008 to 2020 and projection for 2036 in Brazil. Brazil, 2023.

There is a growing trend in total indirect costs. The cost in both sexes increase and continued to increase (Fig. 2) for the 2036 projection.

## Discussion

This study aimed to estimate the costs of obesity by leveraging three databases, spanning from 2008 to 2020, and to project these costs up to the year 2036. The evaluated population comprised individuals aged 25–84 years, with the exception of the indirect costs analysis, where the maximum age considered was 65 years.

The results showed that the direct and indirect costs of obesity attributed to the consumption of sugar-sweetened beverages were in the order of US$ 46.3 million for the sexes and age groups under study. In addition, the results of this study demonstrate that greater attributable burdens of sugar-sweetened beverages were observed in younger individuals and that these decreased with advancing age, with an increase in the age groups over 75 years.

Our findings are consistent with and support previous research conducted in Brazil and other countries on both the health effects of sugar-sweetened beverage consumption and the costs to healthcare systems and the economy^3,35–37^. The majority of studies assessing the economic impact of obesity have been conducted in high-income countries. However, it is now recognized that the economic burden of obesity transcends income levels and geographic contexts^3,37^.

Regarding direct costs, the results showed that the expenditure was higher for women, corresponding to more than 87% of total costs. A recent study showed a similar result in which more than 60% of the expenses attributable to obesity were associated with women. This fact can be explained by the excessive consumption of processed and ultra-processed foods at the expense of minimally processed and fresh foods, by the high prevalence of obesity, and the higher relative risk of certain outcomes, particularly cardiovascular diseases, in this population^17,22^.

The consumption of ultra-processed products directly affects the individual and may harm their health. In the case of sugar-sweetened beverages, which generally have a high glycemic index and rapid absorption, their intake can favor excessive weight gain, insulin resistance, Type 2 diabetes, alterations in the intestinal microbiota, inflammation and atherogenesis^38–43^. Moreover, studies indicate that the consumption of these beverages is associated with unhealthy lifestyles, such as prolonged television viewing and unhealthy eating behaviors^44,45^. These foods also have an indirect impact by overburdening the health system due to the expenses on treatments and medications for obesity and its associated diseases. Furthermore, they impose a burden on the economy through social costs stemming from the loss of productivity related to absenteeism or premature mortality^22–25^.

This study also shows that in the period between 2008 and 2020, indirect costs imposed a burden of US$ 40 million on the economy due to loss of productivity resulting from premature deaths, affecting mainly men of working age. Recent studies have revealed that the consumption of two or more glasses of sugar-sweetened beverages per day was associated with a 17% higher risk of premature death^46,47^. As a consequence of consuming these beverages, more than 12,700 people die annually in Brazil, and 355,400 years of healthy life are lost due to premature death and disability, as indicated by the Disability-Adjusted Life Year (DALY) metric; these are outcomes that could potentially be avoided^48^. One of the possible explanations is related to tax disparities that affect the production, processing, and marketing stages, favoring the production chain of ultra-processed products, making them cheaper and more accessible, and making healthier foods such as rice, beans, fruits, vegetables and legumes more expensive and inaccessible^49^.

The Brazilian tax structure disproportionately favors commodity and ultra-processed product manufacturers, while placing a greater burden on family agriculture. This is exemplified by the Manaus Free Trade Zone, which hosts the largest production facilities for concentrated syrup used in sugary drinks. Businesses operating within this zone are entitled to significant tax benefits, including reductions or exemptions from various taxes such as the Corporate Income Tax (IRPJ), Contribution to the Social Integration Program and Formation of Public Servants' Assets (PIS/Pasep), Contribution to the Financing of Social Security (COFINS), Tax on the Circulation of Goods and Services (ICMS), and Import Tax (II). For the Tax on Industrialized Products (IPI), there is not only an exemption but also the granting of tax credits equal to the amount that would theoretically have been paid for this tax^49^.

The prevalence of obesity has increased worldwide and more rapidly in recent decades. In this study, we observed that men in the age group between 35 and 44 years had a higher prevalence of obesity, reaching about 25%. These findings differ from the worldwide prevalence data, which show a higher prevalence in women than in men^3^. According to the projection made for 2036, a prevalence of around 30% is expected for both sexes, similar to the projection made by the World Obesity Federation, an international organization focused on the reduction, prevention, and treatment of obesity, which predicts that Brazil by 2030 should see almost 30% of its adult population with obesity^3^.

The excessive consumption of sugar-sweetened beverages is known to have an association with overweight and obesity in childhood and adulthood^48^. In Brazil, as in other Latin American countries, sugar-sweetened beverages are popular and consumed in excess. The intake of products high in sugar is considered one of the leading contributors to the prevalence of overweight and obesity^21^.

POF data from 2008–2009 show an inverse association between the price of ultra-processed products and the prevalence of overweight and obesity. A 1% increase in ultra-processed food prices has been shown to reduce the prevalence of obesity by 0.59%, especially in people with lower incomes^49^, suggesting that the taxation of these foods can be a useful tool to help reduce chronic diseases. The taxation of sugar-sweetened beverages generates an increase in prices, which could be a way to discourage their purchase and consumption, in addition to encouraging companies to reformulate their products to reduce the sugar content. It would also contribute to raising tax revenues, which can then be invested in public health programs in the most diverse areas, such as healthcare, the economy and environment^47^.

It is worth noting in this context that obesity is not an individual problem, but the result of the predominance of production chains that invest in and stimulate the production of ultra-processed foods and beverages as well as marketing strategies that seek to influence the choices and preferences of the population towards greater access to ultra-processed products at the expense of fresh and minimally processed foods^47^. As such, cost-effective measures should be developed and implemented urgently to promote healthy eating habits, taking into account the commercial and political determinants of obesity to contribute to the fight against the obesity epidemic.

Understanding the costs attributable to a disease facilitates the prioritization and development of preventive measures, not only at the individual, family, and community levels but also at the environmental level, particularly concerning food systems. Economic analyses enhance epidemiological evidence, serving as a theoretical framework for devising strategies that encourage and reinforce healthy food systems. Furthermore, these analyses assist managers and policymakers in crafting effective public policies and in decision-making processes by providing technical support for comparing the economic implications of various interventions.

Measures aimed at discouraging the consumption of ultra-processed beverages and foods can mitigate health impacts on individuals and, consequently, reduce healthcare and economic costs. Cost-effective strategies could encompass regulatory policies targeting food advertising, including monitoring^50^, limitations of broadcasting hours in the media^51^, punishments for companies that use abusive and misleading strategies, as well as improved nutrition labeling^9^ and taxation of ultra-processed beverages and foods^47^. According to the report “*Health Taxes to Save Lives: Employing Effective Excise Taxes on Tobacco, Alcohol, and Sugary Beverages*,” an increase in the price of sweetened beverages by 50% through taxation could diminish their consumption and, consequently, obesity rates, potentially preventing up to 2.2 million premature deaths over the next 50 years. Additionally, this measure could generate between US$ 700 billion and US$ 1.4 trillion in financial resources^47^.

The Pan American Health Organization has supported taxation on ultra-processed non-alcoholic beverages and this strategy has been adopted in more than 60 territories^49^. Countries such as Mexico, Chile, England, Portugal, Finland, Norway, and France have implemented taxation on these drinks, and research carried out in these countries has shown that this initiative has favored reductions in consumption, increased revenue, fostered the economy due to the change in consumption to other healthier drinks, and stimulated agriculture^47^. Mexico is an example of a globally recognized successful experience, with a 10% increase in the total value of sweetened beverages generating a 7.6% reduction in the consumption of these products^21,52^.

It is worth mentioning that Brazil was one of the first countries to make SMART commitments (Specific, Measurable, Achievable, Relevant and with a fixed Term) as part of the United Nations Decade of Action on Nutrition (UN) 2016–2025. The commitments include preventing the growth of adult obesity, reducing the consumption of sugary drinks by at least 30%, and increasing by at least 17.8% the proportion of adults who eat fruits and vegetables regularly^53^. However, a few years have passed, and not much progress has been made in achieving the proposed goals. In a recent analysis of the Brazilian legislature until 2020, Mariath and Martins^54^ stated that no regulations have been enacted with regard to ultra-processed food industry practices, revealing that there is a major conflict of interest involving the ultra-processed food industries and the Brazilian government. In addition, there is the intense activities of economically powerful interest groups that aim to influence decision-making, making it even more difficult for the goals and objectives established as part of the UN Decade of Action for Nutrition to be achieved^54^.

Studies are needed simulating the taxation of sugar-sweetened beverages and their impact on disease prevalence, the economy and health systems. The taxation of non-alcoholic beverages adapted to the economic, political, and cultural characteristics of each country has been shown to be a cost-effective measure to combat obesity, discouraging the consumption of unhealthy products and generating revenues for the state that can be reinvested in public health policies, enhancing the benefits brought by this measure.

Given the current scenario in Brazilian politics, which aims to discuss the new tax reform, our study is essential. The preliminary text proposes the creation of a selective tax on products and activities detrimental to health and the environment. This tax, intended as a revenue-generating measure, will support the development of policies in health, education, and environmental sectors.

In the analyses of this paper, the costs of supplementary health, i.e., the private network, were not accounted for. It is worth noting, however, that despite the greater share of private spending on the acquisition of health services in Brazil, public spending represents approximately 40% of total expenditures.

Additionally, estimates of direct costs may have been underestimated due to the quality and availability of data extracted from health information systems (SIA and SIH/SUS), which can be limited in many regions of the country. Furthermore, this study did not incorporate the costs of medication from the Popular Pharmacy program or expenditure data from Primary Health Care.

Another aspect that should be mentioned is related to the estimation of indirect costs. In this study, we did not consider other social costs, such as loss of productivity associated with absenteeism or early retirement. There is a high probability that these values are being underestimated, for two main reasons: first, because the variables “country’s state”, “marital status” and “education” remain constant over time. The earlier the individual's death, therefore, the greater the underestimation of their loss of future earnings; and second, Brazil was experiencing an economic crisis that affected employment and, consequently, the population's income in the year 2015, which was used for individual income data in the PNAD.

This study demonstrated that the direct and indirect costs of obesity attributable to the consumption of sugar-sweetened beverages are substantial, affecting public expenditure and leading to social and productivity losses that damage the economy. There is an urgent need for the creation of integrated and intersectoral policies for the prevention and control of obesity that encourage and support the promotion of healthy eating. Strategies that prioritize interventions through fiscal and regulatory measures, such as the taxation of sugar-sweetened beverages, should be reinforced and encouraged to halt the rise of NCDs associated with nutritional factors.

## Data Availability

The microdata used in the study are available in public domain and open access databases. These databases were inserted in the text and in the list of references with the respective URLs, as follows: Brazilian Institute of Geography and Statistics: (https://www.ibge.gov.br/estatisticas/sociais/saude/24786-pesquisa-de-orcamentos-familiares-2.html?=&t=microdados); (https://www.ibge.gov.br/estatisticas/sociais/populacao/9127-pesquisa-nacional-por-amostra-de-domicilios.html?=&t=microdados); and (https://www.ibge.gov.br/estatisticas/sociais/populacao/9126-tabuas-completas-de-mortalidade.html?=&t=downloads. Informatics Department of the Brazilian Unified Health System (DATASUS) for the Outpatient Information System (SIA, specifically, SIA-PA [production subsystem]), Hospital Information System (SIH, specifically, SIA-RD [subsystem of accepted admissions]) and Mortality Information System (SIM, specifically, SIM-DO [death certificate subsystem], all on the same link for access to raw data (https://datasus.saude.gov.br/transferencia-de-arquivos/).

## References

[1] GBD. Global Burden of Disease. Health effects of overweight and obesity in 195 countries over 25 years*. N. Engl. J. Med*. **377**, 13–27. 10.1056/NEJMoa1614362 (2017). 10.1056/NEJMoa1614362 PMC5477817 28604169

[2] GBD. Global Burden of Disease. Global burden of 87 risk factors in 204 countries and territories, 1990–2019: a systematic analysis for the Global Burden of Disease Study. *Lancet*. **396**, 1223–1249. 10.1016/S0140-6736(20)30752-2 (2020). 10.1016/S0140-6736(20)30752-2 PMC7566194 33069327

[3] WHO. World Obesity Federation World Obesity Atlas. Geneva. Available from: https://s3-eu-west-1.amazonaws.com/wof-files/World_Obesity_Atlas_2022.pdf (2022).

[4] BRAZIL. Surveillance of risk and protective factors for chronic diseases by telephone survey. VIGITEL BRASIL 2019. Ministry of Health. Estimates on the frequency and sociodemographic distribution of risk and protective factors for chronic diseases in the capitals of the 26 Brazilian states and in the federal district in 2019. Brasília, DF. Available from: https://bvsms.saude.gov.br/bvs/publicacoes/vigitel_brasil_2019_vigilancia_fatores_risco.pdf (2020).

[5] BRASIL. Plano de Ações Estratégicas para o Enfrentamento das Doenças Crônicas e Agravos não Transmissíveis no Brasil 2021–2030 [recurso eletrônico] / Ministério da Saúde, Secretaria de Vigilância em Saúde, Departamento de Análise em Saúde e Vigilância de Doenças Não Transmissíveis. Brasília, DF. Available from: https://www.gov.br/saude/pt-br/centrais-de-conteudo/publicacoes/svsa/doencas-cronicas-nao-transmissiveis-dcnt/09-plano-de-dant-2022_2030.pdf (2021).

[6] Cordova R (2021). Consumption of ultra-processed foods associated with weight gain and obesity in adults: A multi-national cohort study. Clin. Nutr..

[7] Rauber, F., *et al.* Ultra-processed food consumption and risk of obesity: a prospective cohort study of UK Biobank*. Eur. J. Nutr.* **60**, 2169–2180. 10.1007/s00394-020-02367-1 (2021). 10.1007/s00394-020-02367-1 PMC8137628 33070213

[8] WHO. World Health Organization. Diet, nutrition and the prevention of chronic diseases. Geneva. Available from: https://apps.who.int/iris/bitstream/handle/10665/42665/WHO_TRS_916.pdf;jsessionid=8D1C34440CB532AFA6413EF61D1DCC8E?sequence=1 (2003). 12768890

[9] Jaime, P., *et al.* Diálogo sobre ultraprocessados: soluções para sistemas alimentares saudáveis e sustentáveis. Cátedra Josué de Castro. Nupens/USP. Available from: https://alimentacaosaudavel.org.br/wp-content/uploads/2021/07/Dia%CC%81logo-Ultraprocessados_PT.pdf (2021).

[10] Elizabeth L, Machado P, Zinocker M, Baker P, Lawrencer M (2021). Ultra-processed foods and health outcomes: A narrative review. Nutrients..

[11] Moral AMD, Calvo C, Martinez A (2021). Ultraprocessed food comsumption and a obesity—a systematic review. Nutr. Hosp..

[12] Pagliai G, Dinu M, Madarena MP, Bonaccio M, Iacoviello L, Sofi F (2021). Consumption of ultra-processed foods and health status: A systematic review and meta-analysis. Br. J. Nutr..

[13] Suksatan W (2022). Ultra-processed food consumption and adult mortality risk: A systematic review and dose-response meta-analysis of 207,291 participants. Nutrients..

[14] Askari M, Heshmati J, Shahinfar H, Tripathi N, Daneshzad A (2020). Ultra-processed food and the risk of overweight and obesity: a systematic review and meta-analysis of observational studies. Int. J. Obes..

[15] Chen, X., *et al*. Consumption of ultraprocessed foods and health outcomes: A systematic review of epidemiological studies. *Nutr. J*. **19**, 1–10. 10.1186/s12937-020-00604-1 (2020). 10.1186/s12937-020-00604-1 PMC7441617 32819372

[16] Lane, M. M. *et al*. Ultraprocessed food and chronic noncommunicable diseases: A systematic review and meta-analysis of 43 observational studies*. Obes Rev*. **22**, 1–48. 10.1111/obr.13146 (2021). 10.1111/obr.13146 33167080

[17] IBGE. Brazilian Institute of Geography and Statistics. Household Budget Survey 2017–2018. Analysis of Personal Food Consumption in Brazil. Rio de Janeiro: IBGE; (2020).

[18] Brazil. Ministry of Health. Surveillance of risk and protective factors for chronic diseases by telephone survey. VIGITEL BRASIL 2019. Ministry of Health. Estimates on the frequency and sociodemographic distribution of risk and protective factors for chronic diseases in the capitals of the 26 Brazilian states and in the federal district in 2019. Brasília, DF. Available from: https://abeso.org.br/wp-content/uploads/2020/01/vigitel-brasil-2018.pdf (2020).

[19] Malik, V. S. & Hu, F. B. Sugar-sweetened beverages and cardiometabolic health: an update of the evidence. *Nutrients*.** 11**, 1840–1856. 10.3390/nu11081840 (2019). 10.3390/nu11081840 PMC6723421 31398911

[20] Malik, V. S. & Hu, F. B. The role of sugar-sweetened beverages in the global epidemics of obesity and chronic diseases. *Nat. Rev. Endocrinol.* **18**, 205–218. 10.1038/s41574-021-00627-6 (2022). 10.1038/s41574-021-00627-6 PMC8778490 35064240

[21] WHO. World Health Organization. Guideline: Sugars intake for adults and children. Geneva. Available from: https://www.who.int/publications/i/item/9789241549028 (2015). 25905159

[22] Nilson EAF, Da Silva EN, Jaime PC (2020). Developing and applying a costing tool for hypertension and related cardiovascular disease: Attributable costs to salt/sodium consumption. J. Clin. Hypertens..

[23] Rtveladze K (2014). Obesity prevalence in Mexico: Impact on health and economic burden. Public Health Nutr..

[24] Nilson EAF, Andrade RCS, Brito DA, Oliveira ML (2019). Costs attributable to obesity, hypertension and diabetes in the Unified Health System, Brazil, 2018. Rev. Panam. Salud Public..

[25] Rezende, L. F. M. *et al*. A epidemia de obesidade e as DCNT: Causas, custos e sobrecarga no SUS. Available from: https://rezendelfm.github.io/obesidade-e-as-dcnt/. https://rezendelfm.github.io/obesidade-e-as-dcnt (2020).

[26] IBGE. Brazilian Institute of Geography and Statistics. Household Budget Survey in Brazil, 2008/2009: Anthropometry and nutritional status of children, adolescents and adults in Brazil. Rio de Janeiro: IBGE. Available from: https://biblioteca.ibge.gov.br/biblioteca-catalogo?id=245419&view=detalhes (2010).

[27] Brazil. Ministry of Health. DATASUS database. The National Health System (SUS). Available from: https://datasus.saude.gov.br/transferencia-de-arquivos/.

[28] IBGE. Brazilian Institute of Geography and Statistics. PNAD. Pesquisa Nacional por Amostra de Domicílio. Microdata. Available from: https://www.ibge.gov.br/estatisticas/sociais/populacao/9127-pesquisanacional-por-amostra-de-domicilios.html?=&t=microdados.

[29] WHO. World Health Organization. International Classification of Diseases (ICD). Available em: https://www.who.int/standards/classifications/classification-of-diseases.

[30] IPEA. Institute of Applied Economic Research. Available from: www.ipeadata.gov.br/Default.aspx. Acesso em: 15 jan. 2023.

[31] GBD. Global Burden of Disease. Risk Factors Collaborators. Global, regional, and national comparative risk assessment of 79 behavioural, environmental and occupational, and metabolic risks or clusters of risks, 1990–2015: a systematic analysis for the Global Burden of Disease Study 2015. *Lancet.* **388,** 1659–724; https://www.thelancet.com/journals/lancet/article/PIIS0140-6736(16)31679-8/fulltext (2016). 10.1016/S0140-6736(16)31679-8 PMC5388856 27733284

[32] GBD. Global Burden of Disease. Health effects of dietary risks in 195 countries, 1990–2017: A systematic analysis for the Global Burden of Disease Study 2017. *Lancet*. **393**, 1958–1972. 10.1016/S0140-6736(19)30041-8 (2019). 10.1016/S0140-6736(19)30041-8 PMC6899507 30954305

[33] Micha R (2017). Association between dietary factors and mortality from heart disease, stroke, and type 2 diabetes in the United States. JAMA..

[34] Ywata AXC, Cerqueira DRC, Rodrigues RJ, Lobão WJA (2008). Costs of deaths from external causes in Brazil. Rev. Bras. Biom..

[35] PAHO. Pan American Health Organization. Taxes on Sugar-sweetened Beverages as a Public Health Strategy: The Experience of Mexico. Mexico. Available from: https://iris.paho.org/bitstream/handle/10665.2/18391/9789275118719_eng.pdf?sequence=1&isAllowed=y (2015).

[36] Bardach AE (2023). The burden of disease and economic impact of sugar-sweetened beverages’ consumption in Argentina: A modeling study. Plos One..

[37] Perelli L (2023). Health and economic burden of sugar-sweetened beverages consumption in Brazil. Cad. Saúde Pública..

[38] Martinez Steele E, Juul F, Neri D, Rauber F, Monteiro CA (2019). Dietary share of ultra-processed foods and metabolic syndrome in the US adult population. Prev. Med..

[39] Mendonça RD (2017). Ultra-processed food consumption and the incidence of hypertension in a Mediterranean cohort: The Seguimiento Universidad de Navarra Project. Am. J. Hypertens..

[40] Miclotte L, Van de Wiele T (2019). Food processing, gut microbiota and the globesity problem. Crit. Rev. Food Sci. Nutr..

[41] Schnabel L (2019). Association between ultraprocessed food consumption and risk of mortality among middle-aged adults in France. JAMA Intern. Med..

[42] Srour B, Fezeu LK, Kesse- Guyot E (2019). Ultraprocessed food consumption and risk of type 2 diabetes among participants of the NutriNet-Sante prospective cohort. JAMA Intern. Med..

[43] Zinocker MK, Lindseth IA (2018). The Western diet-microbiome-host interaction and its role in metabolic disease. Nutrients..

[44] Boulos R (2012). ObesiTV: How television is influencing the obesity epidemic. Physiol. Behav..

[45] Rocha LL (2021). Health behavior patterns of sugar-sweetened beverage consumption among Brazilian adolescents in a nationally representative school-based study. Plos One..

[46] Mulle A, Romaguera D, Pearson-Stuttard J, Viallon V (2019). Association Between soft drink consumption and mortality in 10 European countries. JAMA Intern. Med..

[47] OPAS. Organização Pan-americana de Saúde. Taxation of sweetened beverages in Brazil. ACT health promotion Brasília. Available from: https://actbr.org.br/uploads/arquivos/Tributacao-de-bebidas-adocadas-no-Brasil.pdf (2021).

[48] Alcaraz, A., Vianna, C., Bardach, A., *et al.* The hidden side of sugary drinks in Brazil. *Instituto de Efectividad Clínica y Sanitaria.* Buenos Aires, Argentina. Available from: www.iecs.org.ar/azucar (2020).

[49] ACT. ACT technical note for health promotion. For a tax reform in favor of health. Rio de Janeiro. Available from: https://actbr.org.br/uploads/arquivos/NOTA-TECNICA-03-VERSAO-DIGITAL.pdf (2023).

[50] Passos CM, Maia EG, Levy RB, Martins APB, Claro RM (2021). Association between the price of ultra-processed foods and obesity in Brazil. NMCD..

[51] Santana MO (2020). Analysing persuasive marketing of ultra-processed foods on Brazilian television. Int. J. Public Health..

[52] Mytton OT (2020). The potencial health impact of resyticting less-healthy food and beverage advertising on UK television between 05.30 and 21.00 hours: A modelling study. PloS Med..

[53] Colchero MA, Rivera-Dommarco J, Popkin BM, Ng SW (2017). Sustained consumer response: evidence from two-years after implementing the sugar sweetened beverage tax in Mexico. Health Aff..

[54] WHO. World Health Organization. Brazil first country to make specific commitments in UN Decade of Action on Nutrition. Geneva. https://www.who.int/nutrition/decade-of-action/brazil-commitment-22may2017/e (2020).

[55] Mariath AB, Martins APB (2021). Decade of action on nutrition and sugary drinks taxation in Brazil: Where are we?. Cad Saúde Pública..

